# P-1696. Rapid, Comprehensive TB Diagnosis and Resistance Profiling with Deeplex Myc-TB: A Clinically Actionable NGS Approach

**DOI:** 10.1093/ofid/ofaf695.1869

**Published:** 2026-01-11

**Authors:** Marie-Claire Rowlinson, Matthew Schimenti, Patrick Valois, Norma Ordaz-Yuan, Andrew Lin, Justin Ng, Kyle Rhoden, Ramin Khaksar

**Affiliations:** Florida Bureau of Public Health Laboratories, Jacksonville, FL; Florida Bureau of Public Health Laboratories, Jacksonville, FL; Florida Bureau of Public Health Laboratories, Jacksonville, FL; Clear Labs, San Carlos, California; Clear Labs, San Carlos, California; Clear Labs, San Carlos, California; Clear Labs, San Carlos, California; Stanford, San Carlos, California

## Abstract

**Background:**

Timely diagnosis of *Mycobacterium tuberculosis* (MTB) and its drug resistance profile is essential for initiating effective therapy, especially in the context of rising multidrug-resistant TB (MDR-TB) and the availability of new drugs and drug regimens. The Deeplex® Myc-TB assay is a targeted next-generation sequencing (NGS) assay that provides rapid, high-resolution detection of MTB complex species, resistance-associated mutations to 15 anti-TB drugs, and phylogenetic lineage.

**Methods:**

We applied the GenoScreen Deeplex® Myc-TB assay to DNA extracted from 380 clinical specimens from Florida, U.S. Sequencing was performed on an Illumina platform, with results interpreted via the Deeplex bioinformatics pipeline. Manual assay performance was also compared against a fully automated Deeplex® Myc-TB workflow on the Clear Dx™ system.

**Results:**

The assay was performed on 380 primary clinical samples with a success rate of 82.6% following the manual assay process. Testing identified drug resistance in 6.5% of samples tested. Cases of multidrug resistance were < 2% and only one XDR sample was identified. The assay also showed high concordance with phenotypic antimicrobial susceptibility testing by TREK Sensititre MIC method. Comparable data was also generated between the manual versus the automated method. Turnaround time for the manual assay was approximately 4 days and with the automated method this was reduced to 31 hours.

**Conclusion:**

Deeplex® Myc-TB offers a rapid, clinically relevant tool for TB diagnosis and individualized therapy planning. Its ability to guide early treatment decisions makes it particularly valuable for clinicians managing drug-resistant TB at the point of care. Automating this assay not only further reduces turnaround time but also enables less skilled technicians to perform complex TB testing, thereby significantly lowering barrier of entry for any laboratory to contribute to TB diagnostics and stewardship.

**Disclosures:**

All Authors: No reported disclosures

